# Traité sur les Gastralgies et les Enteralgies; ou Maladies de l'Estomac et des Intestins

**Published:** 1828-01-01

**Authors:** 


*Traité sur les Gastralgies et les Enteralgies, ou Maladies Nerveuses de l'Estomac et des Intestins.* Par J. P. T. Barras, M.D. Medecin des Prisons et du Bureau de Charité, &c. Octavo, pp. 330. Paris, 1827. Balliere, Bedford Street, London.

In the 10th Number (fifth volume) of this Series, p. 489—500, we gave a very extended analysis of a paper on the above subject from the same author; in which was detailed Dr. Barras' own case, forming, indeed, the basis of the Memoir. It will be found, that some of the best monographs we possess arose in this way from the personal sufferings of the writers. No verbal description is equal to individual feeling in symptomatology, and, therefore, it often happens, that the pain inflicted on a single person forms a kind of expiation for the multitude. This is particularly the case in respect to stomach affections, to which medical men are very liable, and, from which, the author of the work under review has experienced no small portion of misery. Since the original Memoir was published, he has collected fresh materials, and added them to a more systematic Treatise on this important malady than the Memoir could be said to embrace. We shall endeavour to avoid, or touch very lightly on, the materials contained in the original Memoir, and select, from the present volume, as much of the new matter as possible.

I. Our author takes the definition of Pinel for the class of neuroses, or nervous diseases—" lesion of sense and motion, without inflammation or lesion of structure." This is, perhaps, as good as any other definition. The new, or physiological doctrine is acknowledged to have done great good to medical science, though not unaccompanied by evil in the shape of error. One of these errors is strongly protested against by M. Barras—namely, the doctrine which amalgamates the neuroses with the phlogoses—a doctrine powerfully supported by the late Dr. Parry in this country, but still, a doctrine untenable in theory, and dangerous in practice.

Before the days of Broussais, those nervous affections of the stomach known under the terms gastralgia, gastrodynia, cardialgia, dyspepsia, &c. were treated with bitters, tonics, anodynes, and mineral waters, together with country air and exercise. But the New School could see nothing in this class of disorders, but chronic inflammation of the gastro-intestinal mucous lining, requiring leeches to the epigastrium, gum-water, and starvation. But experience—dire experience, has taught M. Barras (and we venture to say, that it has taught some thousands of others, on both sides of the Channel) that the stomach and bowels may be the seat of an affection purely nervous—that is to say, a lesion of its sensibility, quite independent of inflammation or change of structure, which lesion is rather aggravated than relieved by the rigorous regimen and long-continued depletion employed under the idea that the disease is inflammatory. We shall first introduce the particulars of some of the new cases, by which our author supports his doctrine and practice in gastralgia.

*Case* 1. Madam C. aged 43 years, of very nervous temperament, and subject to pains in her stomach, experienced a severe domestic affliction, in September, 1825. Immediately afterwards, the gastric affection was much aggravated, accompanied by spasms in the chest and sense of suffocation. For these, leeches were thrice applied, mucilaginous drinks prescribed, and the most rigorous regimen enjoined. In November, she became affected with furious delirium, and, in this state, she craved lustily for animal food, and sought to obtain it by main force. M. Barras was consulted, and advised that better nourishment should be allowed. The digestion was distressing at first; but, by gradually habituating the stomach to animal matters, the digestion became easy, and, by the 15th December, the patient could drink a bottle of Bourdeaux wine without inconvenience. With this power of receiving aliment, the strength and flesh returned—her mental aberration disappeared in a great measure, and there is every appearance of complete recovery.

The author remarks that, in this case, it is pretty evident the intellectual disturbance was occasioned by the disorder of the digestive organs. He says, there is but one shade of difference between hypochondriasis and insanity. It is acknowledged, by the best observers, that the *former* is very often dependent on a morbid condition of the digestive apparatus—and, if so, why may not the *latter?* It is true that M. Georget and some other pathologists, place the cause of insanity invariably in the brain. No doubt the *immediate* cause must be in the organ of mind; but this lesion of function or structure in the organ of thought, is very often consequent on disorder in the organs of digestion. In what is called sick headache, the pain is in the head, but the cause is in the stomach or bowels.

*Case* 2. M. Legros, 29 years of age, of nervous temperament, and *Maître d'hôtel* at the Prefecture of Police, had long been subject to stomach-complaints, and had difficult and painful digestion whenever he ate food of a cold or flatulent nature. Many times he vomited up the remains of fruit five or six days after eating it. Four or five years ago, he had had attacks of pain in the epigastric region, which harrassed him for some months, and then went off. In May, 1826, he had a return of these pains, principally after taking food, accompanied by slow and uneasy digestion, eructations, colic, flatulence, and obstinate constipation; but no fever or vomiting. His appetite continued pretty good. Leeches, to the number of ninety-six in all, had been applied, at five different times, to the epigastrium—with gum-water—warm baths—lavements—starvation. After fifty days of this treatment, the physician in attendance was taken ill, and M. Barras was summoned. The patient appeared the very picture of a person who was on the point of dying from hunger! Emaciation had arrived at the last degree of marasmus, and the debility was so great, that the patient could not raise himself in bed. His tongue was moist throughout—white in the middle—red at the sides and extremity—face pale—disgust for drink—vomiting, for some days past, of the gum-water which he had swallowed. Still he had some desire for substantial aliment. The pulse was weak—skin cold—urine aqueous and plentiful—stools very rare—nothing particular about the epigastrium, except that the spine could be easily felt through the abdominal parietes! The *morale* was nearly as much prostrated as the *physique* in this wretched patient.

M. Barras almost despaired of affording relief in such a case, fearing some organic mischief. Nevertheless he cheered the patient with the hope of recovery. Some tender boiled animal food was ventured on, with Brussels biscuit, at first in the smallest quantity, and gradually increased. At the end of twelve days, he could eat the wing of a fowl, or a mutton-chop, and drink some claret and water. The appetite now became so craving, that he required the utmost exertion of his reason to restrain it. Even this abstemious diet was not unattended with some pain and inconvenience, both in the stomach and bowels; but still he gained strength and flesh, and, upon this plan of mild nourishment, he recovered so far as to be able to resume his duties in six weeks. Dr. Marc saw this patient, and can vouch for the truth of the statement.

When Professor Baumes lectured on phthisis pulmonalis at Montpellier, half the students fancied themselves consumptive—Corvisart's writings and lectures caused them to believe they had aneurism of the heart—and, in these days, the Val de Grace Professor has stricken half the medical élèves of France with imaginary gastro-enterites.

"At present, the medical students of the New School dread nothing but chronic inflammation of the stomach. As soon as they feel any uneasiness in the epigastric region, or any symptom of indigestion, they examine their tongues before a glass, or show them to one another—and if they perceive, or fancy they perceive, any redness on the sides or tip, they pronounce themselves affected with *gastro-enterite*. This false idea leads them to the use of leeches in relays—to gum-water—and to acid slops. After a time, this debilitating process engenders a morbid sensibility in the stomach, and the return to solid food is accompanied by pain and inconvenience. They then have recourse to more leeches and other antiphlogistics. By this plan, their stomachs are enfeebled, and their nervous systems so much deranged, that the body and mind act and re-act on one another, so as to render them miserable." 48.

With a very trifling alteration in names, this picture would not inaptly apply, in no small number of cases, on this side of the Channel. Leeches—blue-pill—black draughts, and other measures of the kind, have damaged many a stomach in England, and aggravated, if not engendered, the very disease which the remedies were designed to remove!

*Case 3.* M. N. a provincial physician, 40 years of age, of nervous temperament, was very subject to pains in the stomach, which generally yielded to rhubarb. Being a prisoner in Hungary, during one of these attacks, he applied ice to the epigastrium, and was quickly relieved. He became a zealous convert to the new doctrine. In the month of September, 1824, he was once more seized with gastralgia. He applied leeches to the epigastrium, and put himself on a course of gum-water and slops. He seemed much relieved for a time; but, having been exposed one day to wet and cold, the pains returned with increased intensity, and he considered himself affected with a veritable *gastro-enterite.* Leeches, to the number of 120, were applied to the epigastrium, with all the usual antiphlogistic adjuvants. But, instead of relief, the patient experienced an aggravation of the malady. In this state he came to Paris, in January 1825, and hastened to consult "un medecin physiologiste." The physician confirmed the diagnostic of the patient, but was wise enough to leave off the leeches, and only adhered to the other parts of the plan hitherto pursued. The patient got worse. "The sensibility of the stomach was exalted to such a pitch, that the least particle of food produced great pain, nausea, and insupportable malaise." The tongue became red—the stomach flatulent—the constipation obstinate. The spirits were extremely depressed—the flesh wasted away. The nourishment was still farther diminished. In the mean time, the unhappy patient was dying with hunger, and dared not to eat! In a fit of desperation, one day, he ventured on a bit of chicken. This did not produce much uneasiness. He went into the country, and continued the light animal food, with great benefit. The morbid sensibility of the stomach gradually diminished—the digestion became more easy—the strength and flesh gradually increased—the spirits rose. At this time, M. Barras's Memoir fell into his hands, and he soon recognized his case to be one of gastralgia, rather than gastritis. He accordingly consulted the author of the Memoir, who advised him to pursue the course of light animal food, and in two months he was quite well, with the exception of a slight disposition to hypochondriasis.

*Remarks.* We acknowledge that there is great difficulty sometimes in distinguishing irritation from inflammation—or, in other words, gastralgia from gastritis. In fact, the two states very often co-exist. Extremes approximate in this, as in many other cases; and ultra-depletion, with acid slops, &c. will often augment the uneasiness in the stomach as much as stimulating food. The great art consists in regulating the diet according to the degree of susceptibility in the stomach. We are no advocates for *repletion*, but we are not blind to the injury which is done by *depletion*, in nervous constitutions, where irritation is far more likely to predominate than inflammatory action.

*Case* 4. M. P. aged 26 years, of nervo-sanguineous temperament, and exposed, from his infancy, to constant domestic turmoils and disquiet, applied himself, at a very early age, to intense and long-protracted studies. Before the age of ten years, he became affected with headaches and vertigo—at fifteen, he complained of great irritation in the bladder—and between sixteen and nineteen, he was subject to repeated quinsies. In addition to the above, he had a most capricious stomach—his sleep was bad—and his temper restless and irritable. Up to the Autumn of 1823, however, his digestion had not been much in fault. At that period, his appetite declined—his tongue became furred—pain was felt in the stomach—the pulse was quickened—he had flushings of the cheeks, and sense of constriction about the throat. These phenomena were principally conspicuous during the period of digestion. Wine, coffee, brown, and ultimately white meat, were successively abandoned—and, ultimately, the patient was compelled to live upon soup alone.

Notwithstanding this rigid regimen, M. P. experienced severe pains in the epigastrium two hours after eating, together with obstinate constipation. In this condition the patient continued eight months. In April, 1824, he became affected with a relaxed state of the bowels, the motions being mucous, sanguineous, and containing little or no real fæcal matters. There was much pain in the abdomen, augmented by pressure—discharge of fetid gas from the bowels, with nausea, sense of strangulation, white tongue, quick small pulse, &c. All solid food was prohibited—rice water was ordered to be drunk in abundance—leeches were applied both to the anus and epigastrium, (by which the pains were increased)—emollient injections were prescribed—and one grain of opium was daily given. In three weeks the diarrhœa was removed, and it was succeeded by the original constipation. The sense of hunger was now indomitable, and some rice was allowed. The symptoms were much mitigated. Soup and vegetables, the only food allowed, still produced pain in the stomach, and considerable nervous excitement throughout the system. In July, all the original symptoms returned with aggravation. No cause could be assigned for this, except sulphureous baths, which produced great irritation on the surface of the body. Leeches were again applied, and the patient was twice bled from the arm. The sufferings were increased, and food could not be borne. The disease was now considered to be more nervous than inflammatory, and blisters were applied to the insides of the thighs. The symptoms were still farther exasperated; and, during eight days, there was much fever with excessive pain in the epigastrium. Opium and the warm bath were the only means that gave temporary relief, with the exception of *pressure*, which also solaced the pain. He went into the country in the latter part of August, and there he was obliged to confine himself entirely to light soups. Even these sometimes occasioned great malaise or actual pain. Milk next became his principal nourishment, and of this he took upwards of two pints in the 24 hours. Three months passed in this manner, the patient living sometimes on soup, sometimes on milk. Leeches had been several times applied in this time, but always with increase of the disorder.

About the middle of December he experienced a great exasperation of the complaint—the epigastric pain after eating being insupportable, and to this was added a periodical cephalalgia returning every four or five days, beginning about midday, and lasting till midnight. He had now almost constant fever, nocturnal perspirations, and œdema of the lower extremities. At this time, a vein was opened in the foot, and, for the first time, bleeding gave relief. He went on sometimes better, sometimes worse, till the 3d of March, 1825, when he began to go out in a carriage. He continued to be bled, and to use the warm bath, from time to time. In the Spring of 1826, this unfortunate patient experienced another exasperation of the malady, apparently from having taken a glass of wine. The fever, the furred tongue, the constipation, and other symptoms, became as bad as ever, and leeches were frequently applied, as well as blood taken from the arm. Milk diet was solely employed—and horse exercise was taken. In the months of May, June, and July, the patient ventured on small quantities of fowl and bread. They were digested with pain, and the digestion was almost always accompanied or succeeded by sense of distention in the stomach, difficulty of breathing, acceleration of pulse, and heat of skin. These symptoms were mitigated by decreasing, or entirely omitting, the poultry and bread. Bleeding, however, almost invariably increased the distress. In the course of this Summer, the cold-bath was cautiously tried, and was evidently productive of benefit. In October, 1826, the patient came under the care of Dr. Barras. At this time, his food consisted of small quantities of milk, gruel, chicken, buiscuit, and a few well-boiled vegetables, with water for drink. This diet still produced distention, pain, and even some fever. But his strength was considerable, and he was by no means emaciated. His own remark was as follows:

"My stomach is so capricious, that what will agree one day, will disagree the next; but I always find, that a simple diminution in the *quantity* of my food has more effect in reducing the symptoms than all the leechings or other means that can be devised."

The above case presents a good example of that *morbid sensibility* of the stomach and bowels which seems to constitute the principal feature in dyspeptic affections.* We consider the disease to have been, fundamentally and generally, a gastralgia, rather than a gastritis; but we have little doubt that, occasionally, there was some inflammatory action mixed up with a high degree of nervous irritation in the digestive aparatus.

Dr. Barras has introduced a considerable number of cases from a work by Professor Schmidtman of Berlin, on cardialgic affections, in which the German physician takes nearly the same view of the disease as our author. The plan of M. Schmidtman was generally the exhibition of bitters and tonics, with light plain food, in small quantities, rather than the leechings and slops of the Broussaians. These cases we need not detail. Nor shall we stop to portray the etiology of the disease, as we have given a pretty full view of this in the analysis of the original Memoir.

### THEORY OF NERVOUS AFFECTIONS.

M. Barras conceives that, from an attentive observation of the phenomena of the neuroses, we may divide them into two

* The following passage in a work recently published by Professor Schmidtman, of Berlin, shews the most remarkable correspondence of ideas between the German physician and Dr. Johnson, on the state called morbid sensibility of the stomach.

"Quantum investigando (says he) et cogitando potui assequi, cardialgia primaria (the name which he gives to dyspepsia) semper fundatur *in nimia et immodica ventriculi sensibilitate*."—Summa Observationum, &c. Berlin, 1826.

classes or states—namely, neuroses dependent on irritation or excess of tonicity—and, neuroses dependent on debility, or defect of tone in the nervous system, in the same way that we have inflammations of a *sthenic* and of an *asthenic* character—and hæmorrhages of an active and passive nature. He denominates the former "neuroses *per erethismum*"—the latter, "neuroses *per atoniam*." Tetanus is adduced as the prototype of the former, (tonic neurosis,) and paralysis as that of the latter—or atonic neurosis. The one class is characterised by *exaltation* and disorder of the sensibility, and also of the functions of the nervous system—the other, by depression (affaiblissement) and trouble of the sensibility and functions.

But, as Nature always proceeds by gradual steps, and never suddenly, so, between erethism and atony, there are innumerable shades, till, at length, the two extremes meet and intermingle. But it is to be remembered, that *debility* of the nervous system is very frequently accompanied by *irritability* of the same system, a circumstance that embarrasses us much in the application of remedies. Hence the danger of using stimulants in cases of debility of the stomach, for example—since the morbid sensibility of the same organ may thus be greatly exasperated. "Alors la maladie ne parait consister que dans une *mobilité* extraordinaire de l'appareil sensitif, et elle n'a, pour principal symptôme, que *l'aberration de la sensibilité* et des fonctions de cet appareil." In short, it comes to that state denominated by Dr. Johnson, "MORBID SENSIBILITY"—a condition seldom absent in this class of maladies :—a condition which, as often combining the elements of atony and irritability—of phlogosis and neurosis, demands a mixed or alternated treatment, according as one state or other happens to predominate. It is incredible the mischief that is done by *routine* practice in this class of human maladies! The drastic purgatives, the fiery tonics, and the long continued mercurials, are the bane of dyspepsia—and even where the medicines are proper, the inattention to diet, both by patient and physician, often renders fruitless the whole range of the Pharmacopœia. Well may Dr. Barras observe, that the treatment of dyspepsia "est hygiénique plutot que medicinal."

The author before us remarks that, the precept laid down by most medical men, "to eat little and often," is an error into which he himself fell—and to his cost.

"In this complaint," says he, "it frequently happens that a pressing desire for food takes place a few hours after a reasonable repast. Woe to him who indulges this appetite! It is a morbid sense of hunger which ought to be borne with, unless extremely urgent."* It is certainly better to eat at stated periods, of regular duration, than to be frequently satisfying the stomach. At the same time, it is necessary, in these cases, not to use too sparing a diet, even where there is pain or uneasiness after eating, otherwise the stomach will, as it were, prey on itself, and the irritability will be increased. Indeed, it requires all the vigilance of the physician to watch and manage these cases, so as not to err on either extreme."

We consider that our author has verged towards a dangerous point in the following precept.

"In those cases of gastralgia accompanied by appetite, the patient may eat with perfect security, and without fearing gastritis. He ought not to retrench one particle of the usual quantity taken in health, except in some rare cases, where the alimentary matters are rejected—that is, where the sensibility of the stomach is so exalted, that it cannot bear the contact of victuals. Neither the malaise, the weight at the epigastrium, the exasperation of pain after eating, or even the vomiting of watery matters, some time after meals, should deter him from taking food, since these inconveniences are less prejudicial than continual hunger—and of two evils, it is better to choose the lesser."

We certainly should counsel the patient, in such cases, to keep a guard over his appetite, and not eat quite so much as in health, however pressing the desire for food. We agree with the author in the following precept. "Those who do *not* feel this sense of hunger should content themselves with a small quantity of food, but not abstain from it entirely, unless the aliment be vomited up soon after eating. I have found a small quantity of food, under these circumstances, better than entire abstinence." In those inordinate appetencies, entitled bulimiæ, he cautions the patient against eating to satiety. "In all cases, it is best to eat at stated periods, and to confine the meals to three in the 24 hours."

The quantity, as well as the quality, of our food, in gastralgic affections, is to be strictly attended to. Chicken broth, with rice, barley, or biscuit, is the best species, according to our author, to begin with, and then to gradually ascend to mutton, and even beef, carefully watching the effects. Dr. Barras seems to have a great partiality for rice and maize, but to dread some other of the farinaceæ, as salop, tapioca, &c. He does

---

* This ravenous desire for food, so soon after eating, is a strong proof of "morbid sensibility" in the stomach. This organ cannot bear the presence of food long enough to be perfectly digested, and the consequence is, that the food is passed into the duodenum in a crude state, and due *assimilation* is never performed. Hence the patient wastes in flesh, and decays in strength, while he eats more than a person in health.—*Rev.*

not object to gruel and panada. Dr. B. is inordinate in praise of sugar, as being "veritablement l'ami des nerfs." We have no objection to a moderate proportion of this substance, against which an unfounded prejudice exists on this side of the Channel. Tender and well-boiled vegetables may be ventured on, and continued, unless they produce flatulence or acidity. Asparagus, sea-cale, cauliflower ,young turnips, may be tried when the patient is tired of stale bread and biscuit.

For drink, our author advises water, just coloured with claret or old Burgundy. "In those numerous cases where it is impossible to say whether erethism or atony most prevails, and where we see only proofs of morbid sensibility of the stomach, we ought to begin with the very lightest species of food, in small quantities, and gradually ascend in the scale of diet, according as the digestive power of the stomach improves, and its morbid sensibility diminishes."

He very properly remarks, that we can only lay down general rules, in our dietetics, subjeet to numerous exceptions, varying with the idiosyncrasies of different patients.

Epigastric leeching, Dr. B. confines to cases where the patient is robust, where eruptions or evacuations have been suppressed, and where there are strong symptoms of gastritis, rather than gastralgia. To the vegetable acids and gum-water, so useful in chronic inflammation of the mucous membrane of the stomach, he decidedly objects in cases of dyspepsia.

The morbid sensibility of the gastric and intestinal nerves being once subdued, or at least allayed, we may have recourse to light bitters, and ultimately tonics. But our author very prudently warns the practitioner against the too early use of these medicines, by which thousands of stomachs in these kingdoms are ruined! "Mais, si une longue medication adoucissante entretient et perpetue les maux des nerfs, il y a aussi du danger à passer tout d'un coup à l'emploi des fortifians; ce passage subit et sans gradation ne manque pas de renouveler l'irritation nerveuse :—Il pourrait même enflammer l'estomac."

The author touches on various points of hygiene, as connected with the treatment of gastralgia. He strongly recommends the air of the country, and hints at the good effects of travelling ; but he is evidently unacquainted with the powerful influence of this last species of exercise and recreation on the digestive organs. The French, indeed, are bad travellers—at least they have not half the passion of the English for this agreeable mode of life; nor do they seem to enjoy the natural beauty or majesty of scenery like their insular neighbours. Be this as it may, the man of opulence, or even moderate means, who labours under any of the more severe forms of gastric disorder—and God knows these forms are numerous enough, does a great injury to himself, if he tries not the effect of travelling for its cure, especially among the mountains of Scotland, Wales, or Switzerland. But, as we shall take another opportunity of laying before the profession some more extended observations on this subject, we say no more at present.

